# Peptide Fractions Obtained from Rice By-Products by Means of an Environment-Friendly Process Show *In Vitro* Health-Related Bioactivities

**DOI:** 10.1371/journal.pone.0170954

**Published:** 2017-01-26

**Authors:** Maura Ferri, Jürgen Graen-Heedfeld, Karlheinz Bretz, Fabien Guillon, Elisa Michelini, Maria Maddalena Calabretta, Matteo Lamborghini, Nicolò Gruarin, Aldo Roda, Axel Kraft, Annalisa Tassoni

**Affiliations:** 1 Department of Biological Geological and Environmental Sciences, University of Bologna, Bologna, Italy; 2 Fraunhofer Institute for Environmental, Safety, and Energy Technology UMSICHT, Oberhausen, Germany; 3 Cell Culture Laboratory, Sterlab, Vallauris, France; 4 Department of Chemistry "G. Ciamician", University of Bologna, Bologna, Italy; 5 Carminia snc, Bologna, Italy; Kaohsiung Medical University, TAIWAN

## Abstract

Recently, the isolation of new health-related bioactive molecules derived from agro-food industrial by-products by means of environment-friendly extraction processes has become of particular interest. In the present study, a protein by-product from the rice starch industry was hydrolysed with five commercial proteolytic enzymes, avoiding the use of solvents or chemicals. The digestion processes were optimised, and the digestates were separated in fractions with four different molecular weight ranges by using a cross-flow membrane filtration technique. Total hydrolysates and fractions were tested *in vitro* for a wide range of biological activities. For the first time rice-derived peptides were assayed for anti-tyrosinase, anti-inflammatory, cytotoxicity and irritation capacities. Antioxidant and anti-hypertensive activities were also evaluated. Protamex, Alcalase and Neutrase treatments produced peptide fractions with valuable bioactivities without resulting cytotoxic or irritant. Highest levels of bioactivity were detected in Protamex-derived samples, followed by samples treated with Alcalase. Based on the present results, a future direct exploitation of isolated peptide fractions in the nutraceutical, functional food and cosmetic industrial fields may be foreseen.

## Introduction

The scientific community has become increasingly interested in the valorisation of industrial by-products, with the double aim of reducing waste volume and of recovering added value products. Of particular importance is the re-cycling and valorisation of agro-food industrial by-products as feedstocks for the isolation of bioactive molecules that could be exploited in the nutraceutical, functional food and cosmetic markets, all rapidly evolving sectors always in need of developing new classes of products. One of the most important market trends is the use of new bio-based ingredients obtained by environment-friendly extraction processes and tested by methods that do not imply the use of animals [1]. Therefore, research on plant sources and screening of raw materials has been fostered over the last decade, especially in projects of applied research involving both research performers and small and medium enterprises.

Rice by-products have been suggested as a cheap, renewable and abundant source of antioxidant compounds and bioactive peptides [2]. Rice is a fundamental crop and a staple food for about half of the world’s population and its manufacturing industry produces large volumes of co- and by-products, which are generally under-valued and under-utilised. The two main rice co-products are bran and broken rice. The latter contains about 80% starch and 8% proteins and is generally used by the starch industry to extract powder and crystal starch obtaining proteins as the main by-product [3]. Currently, these residues are mostly used as animal feed given the poor protein solubility [3] even if a few recent studies indicated these proteins as a valuable source for the recovery of antioxidant peptides following whole cell or proteolytic enzyme treatments [4, 5]. In general, antioxidant peptides from plant sources are considered safe and healthy compounds with low molecular weight and different biological activities. They are easily absorbed by the small intestine and, consequently, bioavailable in the human fluids [2, 6]. Usually, plant-derived functional peptides contain 2–20 amino acid residues and may show not only antioxidant properties but also a wide range of other biological activities such as, opiate-like, mineral binding, immunomodulatory, anti-microbial, hypocholesterolemic, anti-hypertensive [2, 6] actions. Rice is known to have nutritional, hypoallergenic and healthy properties, which are retained by peptides derived from rice starch by-products. Up to now, only two studies were published on rice starch by-product re-utilisation for peptide production and some peptides with antioxidant activity have already been isolated by using proteolytic enzymes and *Bacillus* sp. strains [4, 5].

In the present study no solvents or chemicals were used and several bioactivities other than antioxidant capacity were assayed, such as anti-hypertensive, anti-tyrosinase (first demonstration for plant protein hydrolysates) and anti-inflammatory actions, while cytotoxic and irritation effects were not detected. In fact, the aim of the present work was to set up, optimize and scale up an environment-friendly process for the hydrolysis of protein by-products derived from the rice starch industry. Different functional peptide fractions were produced which showed a range of biological activities, suggesting their potential to be exploited as ingredients in the nutraceutical, functional food and cosmetic fields.

## Materials and Methods

### Rice starch by-product: source and characterization

Liquid rice by-product, derived from the industrial starch production from Italian broken rice (composed of kernels of several *Oryza japonica* varieties), was obtained from a local company (Amideria il Cervo Srl, Monterenzio, Bologna, Italy). The by-product, generated at a volume of 120–150 m^3^/day, appeared as a water-based slurry with solid particles. The by-product was certified by the supplier company as GM rice free, with *Escherichia coli*, *Salmonella*, yeasts and moulds not present or below the limit of detection and containing less than 40 CFU/g aerobic bacteria.

Eleven by-product samples (coming from the processing of different initial broken rice batches and/or from single production cycles starting from the same batch) were collected and stored at -20°C before and after analyses. The pH was measured and the solid matter fresh weight was calculated after centrifugation for 10 min at 5000 rpm at room temperature. The percent of dry weight over fresh weight was calculated by weighing 1 g of solid matter (pellet after centrifugation) after drying at 80°C for 2 days. Protein amount was quantified [7] both in the total by-product and in the supernatant. Residual starch analyses were performed on the total by-product using the STA-20 Starch (GO/P) Assay Kit (Sigma-Aldrich, Milan, Italy). At least four biological replicates were analysed for each parameter.

### Protease treatments

The screening of different proteolytic enzymes and the development/optimisation of the digestion process conditions were performed in 15 mL final volume reactions. Initially, five different commercial proteases were tested at 1 U/g (1 enzyme unit per gram of protein) enzyme/substrate ratio (E/S), and 2 hour incubation time at the following pH and temperature optimal enzyme conditions according to literature or to manufacturer’s instructions: Neutrase^®^ 0.8L (neu, pH 7, 50°C, Novozymes A/S, Denmark), papain (pap, pH 6.5, 50°C, Sigma-Aldrich, Milan, Italy), Alcalase^®^ 2.4L (alc, pH 7, 60°C, Novozymes A/S, Denmark), Protamex^®^ (pro, pH 8, 60°C, Novozymes A/S, Denmark), Flavourzyme^®^ 500L (flav, pH 7, 60°C, Novozymes A/S, Denmark). The digestions were stopped by boiling the samples in a water bath for 10 min to inactivate the enzymes. Following optimisation experiments mainly focused on E/S ratio testing 0.05, 0.5, 1, 2, and 10 U/g. In all experiments, the final pH was checked and the total digestates and the supernatant fractions (obtained after centrifugation for 10 min at 5000 rpm at room temperature) were analysed to assess total protein content [7] and the molecular mass distribution of proteins and peptides (mono-dimensional SDS-PAGE, 16% w/v acrylamide) [8]. At least two biological replicates, with two technical replicates each, were performed for all the digestion conditions.

The three best digestion processes and the not digested control at room temperature (ND) were scaled-up in 500 mL flasks containing 240 mL of final volume reaction (six replicates for each treatment) using the following conditions: 1) 0.5 U neu/g, pH 7, 50°C, 2h; 2) 0.5 U alc/g, pH 7, 60°C, 2h; 3) 0.5 U pro/g, pH 8, 60°C, 2h. pH, dry weight, protein content and SDS-PAGE molecular mass distribution were analysed.

### Fractionation process

The liquid supernatants (about 850 mL each) of the scaled-up alc, neu and pro digestions were sub-fractionated by cross-flow membrane filtration to isolate peptide samples having different molecular weight ranges. The peptide fractionation was performed with a Sartorius Slice 200 Benchtop system (Sartorius-Stedim, Melsungen, Germany) using polyethersulfone (PESU) cassettes with a cut-off of 0.2 μm (0.02 m^2^), 8 kDa (0.04 m^2^), 5 kDa (0.04 m^2^) and 1 kDa (0.04 m^2^) molecular weight. The filtration was performed on ice using a flow rate of 190 mL/min with a maximum trans-membrane pressure of 2 bar. The filtration was stopped when the retentate volume reached 50 mL.

### *In vitro* biological activities assessment

Antioxidant capacity was measured by the ABTS (2,2-azino-bis-3-ethylbenzothiazoline-6-sulfonic acid) assay previously optimised for cereal samples [9] with results expressed as ascorbic acid (AA) equivalents/L. Anti-hypertensive capacity was evaluated by the angiotensin converting enzyme (ACE) inhibitory test [10] with minor modifications. ACE enzymatic activity was assayed by monitoring the amount of hippuric acid derived from the hydrolysis of the substrate hippuryl-histidyl-leucine in the presence of the sample. Pyridine and benzene sulfonyl chloride (40% and 20%, respectively, on the final assay volume) were added and the absorbance of the developed yellow coloured solution was measured at 410 nm. The percent inhibition curves were plotted using a minimum of five increasing concentrations for each sample and the IC50 value was calculated. Anti-tyrosinase activity was assessed by an optimised tyrosinase inhibition assay [11]. The kinetic of brown colour formation was evaluated (490 nm absorbance measurement) in a reaction containing 10 U of tyrosinase and 2 mM L-DOPA in the presence of the sample. The results were expressed as kojic acid (KA, a well-known tyrosinase inhibitor) equivalents/L by means of a dose-response calibration curve (between 1 and 10 μg of KA). A bioluminescent cell-based assay for anti-inflammatory activity was performed using human embryonic kidney HEK293 cells (ATCC, American Type Culture Collection, Manassas, VA, USA) routinely grown in Dulbecco Modified Essential Medium (DMEM high glucose 4.5 g/L, GE Healthcare, Milan, Italy), supplemented with 10% (v/v) fetal bovine serum, 2 mM L-glutamine, 50 U/μL penicillin, and 50 μg/mL streptomycin. The day before transfection, HEK293T cells were plated in a 24-well plate at a density of 8 x 10^4^ per well. Cells were co-transfected with plasmid pGL4.32[luc2P/NF-κB-RE/Hygro] containing five copies of the NF-κB response element (NF-κB-RE) driving transcription of the luc2P reporter gene (Promega, Madison, WI, USA), and plasmid pmcherryPRET9 expressing the fluorescent protein mcherry-C1 (Clontech, Mountain View, CA, USA) and a red thermostable *P*. *pyralis* luciferase mutant [12], obtained by standard molecular biology procedures. Co-transfections were performed by using FuGENE®HD according to the manufacturer's instructions and incubated at 37°C with 5% CO_2_ for 24 h. Forty-eight hours post-transfection, cells were co-incubated for 20 hours with 500 μL of fresh medium containing sample (1:20 dilution) and 20 ng/mL TNFα. After incubation at 37°C, cells were detached with trypsin-EDTA 1X in PBS, resuspended in 100 μL PBS 0.1 M pH 7.5 and then transferred to black 96-well microplates. Fluorescence (FL) and bioluminescence (BL) measurements were performed with a Varioskan™ Flash Multimode Reader. The FL signal was obtained exciting samples at 570 nm and acquiring the signal at 610 nm, while the BL signal was acquired with band pass green and red filters after injection of the substrate BrightGlo [13]. Statistical analysis was performed by using one-way Anova with *p* < 0.05 accepted as significant. Basal activation of NF-κB or activation with 20 ng/mL TNFα were used to calculate fold of induction of treated cells *vs* control cells. Cytotoxicity and irritation tests were performed on Sterlab Reconstructed Human Epidermis (RHE). For cytotoxicity evaluation, human tissues were placed in a 24-well plate with medium and exposed topically to pure samples for 24 hours at 37°C. After washing with PBS, a cell viability test was performed. For irritation evaluation, human tissues were placed in a 24-well plate with medium and each tested substance was topically applied for 42 min at room temperature. Exposure to the substance was followed by rinsing with PBS and mechanical drying. RHEs were transferred to fresh medium and incubated at 37°C for 42 additional hours. Then a cell viability test was performed. For each test the cell viability was assessed by incubating the tissues for 3 hours with 0.3 mL 3-(4,5-dimethylthiazol-2-yl)-2,5-diphenyltetrazolium bromide (MTT) solution (0.5 mg/mL). Formazan crystals were extracted for 2 hours at room temperature using 1.5 mL isopropanol and quantified by spectrometry at 550 nm absorbance. SDS 0.1% (w/v) and PBS were used as positive and negative controls, respectively. For each treated tissue, the cell viability was expressed as percentage of the mean negative control tissues. A cell viability above 50% indicated the not toxicity or the not irritancy potential of the tested substance.

## Results and Discussion

### Rice starch by-product characterisation

The protein by-product feedstock, obtained from industrial rice starch production, consisted of a water-based slurry with solid particles in suspension. Eleven rice by-product samples were characterised and analysed for different parameters (Table 1). The pH value was always around neutrality. On average, solid matter content was 7.5% of the sample volume, with a dry weight/fresh weight ratio of 22.8%, corresponding to 19.3 g/L. Proteins were mostly present in the solid part of the by-product, while almost absent in the liquid phase (below 5% of total sample proteins). Residual starch was present in variable amounts, depending on the industrial extraction efficiency and on the specific cycle of production (Table 1).

**Table 1:** Characterisation of initial rice by-product.

		Average	Range
**pH**		6.7 ± 0.5	6.1–7.3
**Solid matter**	% solid matter / volume	7.5 ± 2.4	3.5–12.5
	% dry weight / fresh weight of solid matter	22.8 ± 4.9	12.0–29.7
	dry weight (g/L)	19.3 ± 8.2	6.7–31.3
**Protein amount**	total sample (g/L)	6.0 ± 4.6	1.4–17.7
	supernatant (g/L)	0.3 ± 0.3	0.0–0.7
**Residual starch**	total sample (g/L)	1.6 ± 1.7	0.1–5.2
	% in the solid matter	7.0 ± 5.3	0.7–16.8

Average data ± SD (n = 11 to 44 depending on the analysis type) and range for each parameter were measured on eleven different by-product samples.

### Protease hydrolysis: enzyme selection and E/S optimisation

According to the literature, one or more steps of rice by-product preparation, such as defatting the starting material by hexane extraction and/or removing phenolic compounds by ethanol washing, are usually performed prior digestion [4, 14–16]. In the present study, it was chosen to not apply any of these steps as the final aim was to optimize an environment-friendly process for application in nutraceutical and cosmetic fields, in which the use of solvents is strongly discouraged.

**Fig 1:**
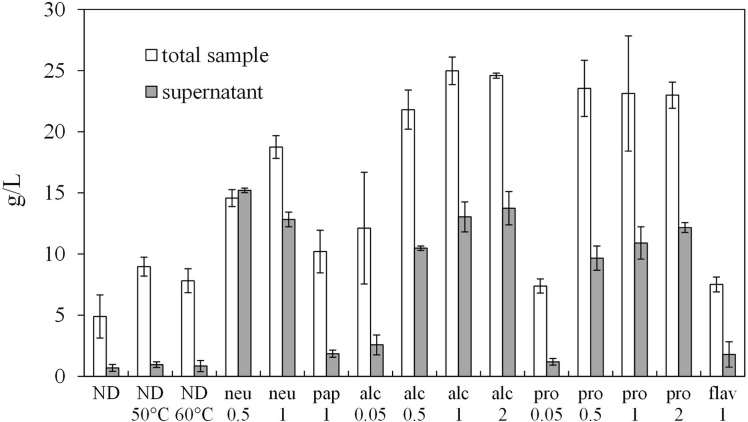
Protein quantification on total and supernatant samples. Enzymatic hydrolyses (2 hours) with Neutrase (neu), papain (pap), Alcalase (alc), Protamex (pro) and Flavourzyme (flav), were performed. Numbers under the enzyme abbreviations indicate the E/S ratio in U/g (unit of enzyme per g of protein). Results are expressed as g of protein per litre of digestate (g/L) ± SD (at least n = 4).

Fig 1 summarises several by-product protease digestion experiments.

Protein rice by-product, without any initial pre-treatment, was first digested for 2 hours using five commercial proteases (Neutrase (neu), Papain (pap), Alcalase (alc), Protamex (pro) and Flavourzyme (flav)) in 15 mL final reaction volume, at an E/S ratio of 1 U/g (units of enzyme per g of proteins). Protein yield was quantified both in the total digested sample and in the supernatant after centrifugation and compared with the not digested controls performed at room temperature (ND) or incubated at the same temperature as the enzymatic digestions (ND 50°C, ND 60°C). In the not digested samples, proteins were present on average at a concentration of 6.7 g/L, while they were at a much lower concentration in the supernatants (below 0.66 g/L) (Fig 1), confirming that almost all proteins are present in the solid matter of the by-product. When comparing the three ND controls, a slight protein increase in the total sample was observed when the by-product was incubated at 50°C or 60°C, probably due to the temperature increase, while no differences were observed among the control supernatants. A significant increase of measurable proteins, both in the total sample and in the supernatant, was detected after digestion with neu, alc and pro enzymes (at 1 U/g) (Fig 1). On the contrary, flav and pap did not induce peptide release as efficiently as the other three enzymes (Fig 1) and, therefore, these enzymes were not further considered. An increase of 18.8, 19.1 and 15.9-fold was measured in the supernatant of neu, alc and pro, respectively, when compared to ND sample (Fig 1). The different peptide yields obtained with the five treatments (Fig 1) could be explained by differences between the proteases used: the three most efficient ones (alc, neu and pro) are all serine-endopetidases, pap is a cysteine protease and flav is a protease/peptidase with both endoprotease and exopeptidase activities. Serine proteases, named for the nucleophilic Ser residue at the active site, constitute almost one-third of all proteases [17]. At least four classes of ser-proteases with variable specificities can be distinguished [17], but it is reasonable to expect that alc, neu and pro digestates are more similar among each other than pap and flav. Probably pap and flav specific protein substrates were less present in rice by-product than those specific for ser-protease. Moreover, flav has also exopeptidase activity and should, therefore, release more free amino acids (which were not quantifiable by Lowry assay) than the other treatments, leading to a lower peptide yield.

**Fig 2:**
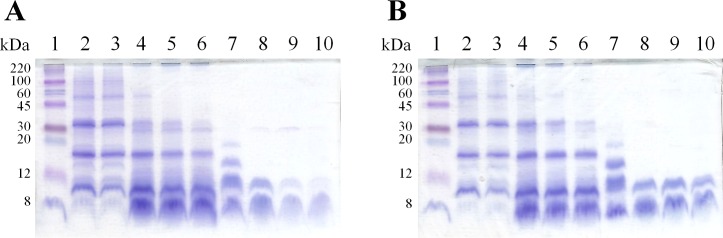
Mono-dimensional SDS-PAGE (16% w/v acrylamide) protein separation of digestates obtained with different E/S (U/g) ratios. (**A**) Alcalase and (**B**) Protamex hydrolysates after 2 hours of incubation. Loaded volume: 3 μL of total samples, 25 μL of supernatants. Loading scheme: lane 1) molecular weight markers; 2) ND, not digested total sample at room temperature; 3) ND 60°C, not digested total sample 2h at 60°C; 4) 0.5 U/g, total sample; 5) 1 U/g, total sample; 6) 2 U/g, total sample; 7) ND 60°C, not digested supernatant 2h at 60°C; 8) 0.5 U/g, supernatant; 9) 1 U/g, supernatant; 10) 2 U/g, supernatant.

Peptides smaller than 12 kDa in size were detected in the mono-dimensional SDS-PAGE separation of total samples and supernatants (Fig 2). Although a large portion of rice proteins is not soluble in water [18], the protein slurry seemed to be hydrolysed by proteases and peptides were recovered in the liquid phase (Fig 2A and 2B, lanes 8 to 10), which may represent an important advantage for their recovery in a future industrial process. On average, rice endosperm proteins are composed of 66–78% glutelins, 9.6–10.8% globulins, 3.8–8.8% albumins, and 2.6–3.3% prolamins [18]. Recently, Dei Più and colleagues [5], after the hydrolysis of a similar rice starch by-product (using alc, neu or a *Bacillus pumilus* strain), identified several peptides, mostly derived from glutelins.

The present results (Fig 1) are in agreement with recent data from Uraipong and Zhao [14] regarding the hydrolysis of rice bran, in which pro enzyme was found to be the most efficient, followed by alc, neu and flav. A low proteolytic efficiency of flav and neu in hydrolysing rice proteins was also reported by Zhao et al. [16], who conversely obtained high protein digestion yields with alc or pro. The previously described processes were further optimised by testing additional E/S ratios (Fig 1). In alc and pro digestions, an E/S ratio of 0.05 U/g determined a protein yield decrease in the supernatant of, respectively, 80.3% and 89.1% compared to 1 U/g, while in 0.5 and 2 U/g digestates the protein amount was not significantly different compared to 1 U/g (Fig 1). After enzymatic hydrolysis, with the exception of 0.05 U/g treatments, the total protein content increased up to 4-times with respect to ND, both for alc and pro digestions (Fig 1). In the case of neu digestion, 0.5 U/g led to a 22.3% decrease of protein amount in the total sample with respect to 1 U/g treatment; consequently the 0.05 U/g neu hydrolysis was not performed (Fig 1). The higher protein content generally measured in the digested supernatant with respect to the ND control suggested the presence of amino acids and small molecular weight peptides, which were in fact detected by mono-dimensional gel electrophoresis (Fig 2). As expected, high molecular weight proteins (ND total samples, Fig 2A and 2B lanes 2–3) were hydrolysed in supernatants of digested samples, and peptides under 12 kDa were detected both after alc (Fig 2A) and pro (Fig 2B) treatments. Moreover, as a consequence of the more efficient digestion obtained with increasing E/S ratio, it was observed that alc keeps digesting the released small peptides, which in fact were detected at lower levels in 1 and 2 U/g samples (Fig 2A, lanes 9 and 10) when compared to the 0.5 U/g sample (Fig 2A, lane 8). These results indicate that an increase of E/S ratio seems not necessary when the process is only aimed at peptide release. Since no significant improving differences were observed among the tested E/S ratios, further experiments were performed with 0.5 U/g of protease.

### Process scale up and digestate *in vitro* biological activities

**Fig 3:**
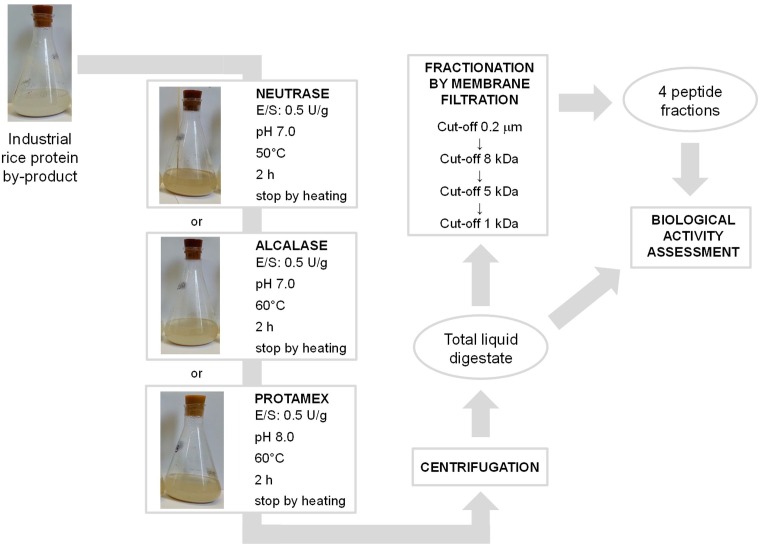
Schematic representation of the proposed process. Rice protein by-product from starch industry was enzymatically hydrolysed by one of the three selected commercial proteases at the defined conditions. The resulting liquid supernatant was fractionated by a sequence of four cross-flow membrane filtrations at different molecular weight cut-offs, leading to the isolation of different peptide ingredients.

Previous results (Figs 1 and 2) indicated that alc, neu and pro (at E/S of 0.5 U/g and 2 hours of incubation) were the most efficient in digesting rice protein by-product and in releasing peptides. These three selected conditions, plus the ND control at room temperature, were scaled up in flasks containing 240 mL of rice by-product final volume reaction (Fig 3).

In contrast to the previous 15 mL-scale reactions, a change of colour was observed in protease-supplied samples (Fig 3) but not in the ND sample. The final brownish hint is probably due to the Maillard reaction, which might occur between some amino acids and reducing sugars present in the by-products. This reaction typically produces brownish molecules, usually responsible for food distinct flavours (e.g. typical flavour of bakery products).

The three supernatants obtained by centrifugation of alc, neu and pro flask digestions were analysed confirming the increase in protein concentration after protease hydrolysis (0.4 g/L in ND, 4.4 g/L in neu and pro and 5.2 g/L in alc) and the release of peptides. To evaluate the influence of the scaled-up reaction volume, 15 mL digestions were also performed, in parallel to flasks at the same conditions and using the same by-product batch. No significant differences were observed between the two reaction volumes. However, in comparison to previous results (Fig 1), slightly lower final protein yields were obtained due to the use of a different initial by-product batch.

ABTS assay results demonstrated an increase in antioxidant activities in the supernatants after flask protease digestion with respect to the control sample (Table 2).

**Table 2:** *In vitro* biological activities of the supernatants collected after scale up of protease digestion in flasks.

Sample	Antioxidant activity (gAA/L)	Anti-hypertensive activity (IC50, g/L)	Anti-tyrosinase activity (gKA/L)	Inflammatory activity (fold induction of NFκB)	Anti-inflammatory activity (fold induction of NFκB with TNFα)	Cytotoxicity (cell viability)	Irritation (cell viability)
ND	0.038 ± 0.001	not detectable	0.046 ± 0.005	1.3 ± 0.4	1.4± 0.5	68.3%	99.8%
neu	0.50 ± 0.01	0.44	0.047 ± 0.005	1.7 ± 0.1	1.3± 0.4	97.7%	89.4%
alc	0.46 ± 0.01	0.56	0.036 ± 0.004	1.1 ± 0.3	1.2± 0.4	102.9%	89.4%
pro	2.31 ± 0.01	0.018	0.318 ± 0.032	not detectable	0.8± 0.6	100.8%	92.1%

ND, not digested samples, Neutrase (neu), Alcalase (alc), Protamex (pro), ascorbic acid (AA), kojic acid (KA). Average data ± SD (at least n = 3).

The best result was obtained with pro supernatant (2.3 g of ascorbic acid (AA) equivalent per L), which showed an almost 61-fold higher antioxidant activity than the ND control. However, it has to be pointed out that other researchers found chymotrypsin or neu enzymes to be best performing in releasing antioxidant peptides from rice by-products [4, 5]. The Angiotensin Converting Enzyme (ACE) inhibitory assay demonstrated that rice by-product digestates had a potential anti-hypertensive capacity (Table 2). The highest percentage of ACE inhibition was again obtained with pro supernatant (83% of inhibition with supernatant diluted 1:10). When expressing the results in IC50, pro sample confirmed to be the most active (IC50 = 0.018 g/L), followed by neu (IC50 = 0.44 g/L) and alc samples (IC50 = 0.56 g/L). ACE inhibitory activity of ND control was not detectable. In general, the pro result is very promising. In fact previous rice seed protein hydrolysated with alc, showed a 7.8-times lower *in vitro* ACE inhibitory activity (IC50 = 0.14 g/L), than the present pro digestate (Table 2), nonetheless producing a significant decrease in systolic blood pressure in spontaneously hypertensive rats after oral administration [19]. The present study demonstrates *in vitro* and for the first time an anti-tyrosinase activity for plant protein hydrolysates (Table 2). The only published data regarding anti-tyrosinase activity of rice were related to phenolic acids and their derivatives and were assessed both *in vitro* [20] and *in vivo* [21]. As observed for antioxidant and ACE-inhibitory capacities, pro hydrolysate was the most active, with anti-tyrosinase activity equivalent to 318 mg of kojic acid per L, about 7-fold higher than in the ND sample. Neu and alc supernatants displayed activities not significantly different from the ND control (on average about 43 mg of kojic acid (KA) per L). Anti-inflammatory activity was evaluated as the capability of samples to inhibit TNFα-induced NF-κB activity in an *in vitro* cell-based assay. All tested fractions showed negligible anti-inflammatory activity (Table 2). These results are in agreement with previous data that correlated rice anti-inflammatory effects with flavonoids or other phenols [22]. The cytotoxicity assay (by means of a Reconstructed Human Epidermis (RHE) model) proved that the three supernatants analysed were not toxic (Table 2). In fact, a mean tissue cell viability >50% is considered not toxic, with all the analysed samples leading to a cell viability between 98 and 103%. The negative and positive controls led to, respectively, 100% and 12.42% cell viability. The irritation test for each supernatant sample also used the RHE model (Table 2). Negative and positive controls led to, respectively, 100% and 3.09% cell viability. A sample that produces a mean tissue cell viability <50% is considered irritant (R38). As the three supernatants led to a cell viability between 89 and 92% they can be considered as not irritant. To our knowledge, the present study is the first scientific report on the use of the RHE model for testing plant protein hydrolysates. In fact, there are only a few papers that report the testing of plant polyphenolic extracts by RHE (such as *Paeonia lactiflora* [23] and *Populus nigra* [24]) for their depigmenting potential. This technique is based on the principle of air-liquid interface human cell culture using chemically defined culture media, resulting in 3D human tissue model. This model allows safety and efficacy tests of new ingredients, avoiding the use of animals. The development and validation of *in vitro* testing models have been rapidly increasing in Europe since 2014, given the requirements of the EU Cosmetic Directive (76/768/EEC) 7^th^ Amendment (2003/15/EC) that prohibits to put animal-tested products on the cosmetic market.

The present data led to the preliminary conclusion that the use of different proteases allows to obtain digestates with different biological activities probably as a consequence of a diverse final peptide composition of the samples. Differences in the distribution of peptide molecular weight and amino acid profile among rice protein hydrolysates obtained with different proteases were in fact recently demonstrated [16]. It was also suggested that the current technologies available to release bioactive peptides from food (e.g., enzymatic hydrolysis to simulate the environment in the gastrointestinal tract, chemical hydrolysis with acids, fermentation with microorganisms on food products) can be modulated to produce specific peptides of interest depending on their intended final use [2].

### Fractionation and biological activity of the isolated peptide fractions

The three total aqueous supernatants, derived from alc, neu and pro flask digestions and containing proteins and peptides, were fractionated by means of four cross-flow filtrations using membranes at different cut-offs. The suspended solid matter was removed by a filtration step using a 0.2 μm pore size membrane. The retentate of this filtration was not further analysed as it contained undigested proteins and high molecular weight peptides, while the permeate was subsequently filtrated through 8 kDa, 5kDa and 1 kDa membranes. After filtration, the 1 kDa permeate was concentrated to a final volume of 50 mL by rotary evaporation (60°C, 1 mbar). The advantage of this separation method is that it employs only physical separation techniques without the use of chemicals, leading to end products that are completely safe and exploitable for nutraceutical or cosmetic applications. A scheme of the proposed process is reported in Fig 3.

Protein content and biological activities of isolated peptide fractions were assessed. The fractions were named as follows: retentates of 8 kDa (R8, molecular weight higher than 8 kDa), 5 kDa (R5, molecular weight between 8 and 5 kDa) and 1 kDa (R1, molecular weight between 5 and 1 kDa) membranes and 1 kDa permeate (P1, molecular weight lower than 1 kDa).

**Fig 4:**
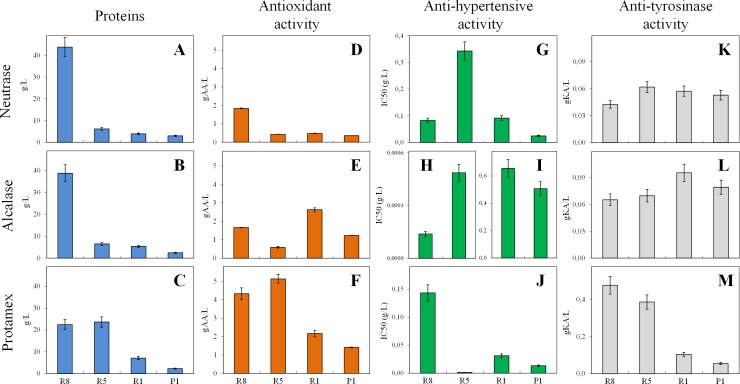
Protein quantification and biological activities of peptide fractions. Protein concentration in g/L (A, B, C), antioxidant activity expressed as g of ascorbic acid (AA) per L (D, E, F), anti-hypertensive activity IC50 expressed as g/L (G, H, I, J), and anti-tyrosinase activity expressed as g of kojic acid (KA) per L (K, L, M) on fractions obtained from Neutrase, Alcalase and Protamex hydrolyses. Data represent the mean ± SD (at least n = 3).

After protein determination, the highest peptide content was in general detected in R8 fractions (Fig 4A, 4B and 4C), with exception of the pro digestion that resulted in almost equal amount of proteins in R8 and R5 fractions (Fig 4C). Differences in molecular weight distribution (determined by protein purification chromatography) among neu, alc, pro and flav rice protein hydrolysates were previously observed by Zhao et al. [16], even though the correlation with functional properties or antioxidant activities was not clarified.

ABTS assay results demonstrated that antioxidant capacity (Fig 4D, 4E and 4F) of the fractions was generally in agreement with protein content (Fig 4A, 4B and 4C). The best results were obtained with pro digestate with all fractions displaying antioxidant activities between 1.4 and 5.1 gAA/L (corresponding to 0.19 and 0.61 gAA/g prot) (Fig 4F). After alc hydrolysis the best result was found with R1 (2.6 gAA/L, corresponding to 0.49 gAA/g prot) (Fig 4E), while neu digestion produced peptides with a lower antioxidant activity (on average 0.7 gAA/L or 0.09 gAA/g prot) (Fig 4D). Each fraction contains many different peptides, with molecular weights in the same range but each with a different sequence and structure. The structure-activity relationship of antioxidant peptides derived from natural proteins has been largely studied but not yet completely clarified [25]. Recently, Wattanasiritham et al. [15] isolated peptides from rice bran treated with papain or trypsin evidencing that antioxidant activities of the hydrolysates were significantly higher compared to untreated proteins. Most of the identified peptides showed a molecular weight between 0.8 and 2.1 kDa, corresponding to 6–21 amino acid residues [15]. In the present work, peptides having a similar molecular weight should presumably be found in P1 and R1 fractions, in particular from pro and alc digestions, which are in fact those displaying the highest antioxidant activity when data were expressed as g AA per g of protein (0.50 and 0.61 gAA/g in alc and pro P1 fractions; 0.49 and 0.30 gAA/g in alc and pro R1 fractions). ACE inhibitory activity assays showed that all fractions had a potential anti-hypertensive capacity (Fig 4G, 4H, 4I and 4J), with alc R8 and R5 having the best potential anti-hypertensive capacity (IC50 values of 1.4·10^−4^ and 4.9·10^−4^ g/L, respectively, Fig 4H) followed by pro R5 and P1 (IC50 of 0.0016 and 0.013 g/L) (Fig 4J). The present results seem very promising when compared with recent published data obtained from rice bran protein hydrolysates [14] which evidenced an average IC50 of 11.3 g/L and 11.8 g/L in samples digested, respectively, with pro and alc which is, respectively, 240 and 41-fold higher (i.e. less active) than the average of the pro and alc fractions here reported (Fig 4H, 4I and 4J). The present data also confirm that alc and pro treatments produce hydrolysates with higher inhibitory activity than neu treatment [14]. It has been suggested that ACE inhibitory action is related to specific structural amino acids (such as proline, lysine or arginine at C-terminus), that short peptides (2–5 amino acids) are the most effective and that the potency of peptides decreases with increasing chain length [26]. Present results obtained with neu and pro samples seem to confirm that fractions containing lower molecular weight peptides (R5, R1 and P1) are in general more active than those containing higher molecular weight peptides (R8) (Fig 4G and 4J). Li et al. [19] also proved that not-hydrolysed rice proteins showed no inhibitory effect on ACE, while alc total digestate exerted a potent *in vitro* inhibitory activity in addition to an *in vivo* antihypertensive effect in spontaneously hypertensive rats after a single oral administration. Anti-tyrosinase activity assessment was also reported for the isolated peptide fractions. Samples obtained after alc digestion showed on average a 45% higher anti-tyrosinase capacity than those obtained with neu digestion (Fig 4K and 4L). Overall, pro R8 and R5 samples were the most active, in particular, their activity was 7.4 and 5.5-times higher than those obtained with alc R8 and R5 samples (Fig 4L and 4M). Instead, when data were expressed as gKA/g of protein, anti-tyrosinase activities of neu- and alc-derived samples seemed to be generally higher in fractions having lower molecular weight peptides.

Sample dilutions with a concentration range of 0.2–20 g/L were also tested for inflammatory activity by a bioluminescent *in vitro* cell-based assay (Table 3). Some fractions showed a significant inflammatory activity, such as those from neu and pro digestions, with fold induction values ranging from 2 to 18. For some of these fractions, characterized by high cytotoxicity in HEK293T cell line, this activity is explained by the well-known role of NFkB in cell survival regulation and, in particular, in apoptotic events. The same dilutions were also tested in the presence of TNFα to point out their possible anti-inflammatory capacity. Unexpectedly, three out of four pro fractions showed anti-inflammatory activity, although these preliminary results were not corroborated by further tests performed using different concentrations of the same fraction to investigate dose-dependent effects. Only the alc P1 fraction showed a concentration-dependent anti-inflammatory activity in the range 0.06–0.2 g/L (data not shown) with a maximum inhibition of 0.25-fold at a concentration of 0.2 g/L. This kind of assay has not been previously reported for rice peptides and provides a suitable and robust tool to rapidly evaluate biological activity of rice peptide fractions. Moreover, the introduction of an internal viability control based on a constitutively expressed reporter protein, allowed to obtain data on cell viability and, consequently, on fraction toxicity. Recently, the specific peptide sequence RGD (arginine-glycine-aspartic acid), detected in rice glutelin storage proteins, has been linked to several bioactive properties, including anti-inflammatory activity and integrin-mediated response. This sequence seems not present in other cereal grain storage proteins (e.g., from wheat, oat and barley), suggesting that rice proteins could have unique beneficial health properties [2]. Cytotoxicity and irritation capacity of fractions coming from alc and pro hydrolysis were tested on the RHE model. All tested fractions resulted not cytotoxic and not irritant (Table 3).

**Table 3:** Anti-inflammatory activity and cytotoxicity and irritation effects of peptide fractions in *in vitro* cell-based systems.

Sample	Inflammatory activity (fold induction of NFκB)	Anti-inflammatory activity (fold induction of NFκB with TNFα)	Cytotoxicity (% viability)	Irritation (% viability)
neu R8	6.9 ± 1.3	not detectable	77.04	94.55
neu R5	10.1 ± 3.2	not detectable	67.71	90.60
neu R1	7.9 ± 0.9	not detectable	96.91	93.53
neu P1	2.2 ± 0.3	1.3 ± 0.2	69.94	91.43
alc R8	2.9 ± 0.3	1.3 ± 0.1	109.33	94.55
alc R5	1.3 ± 0.4	1.5 ± 0.1	99.17	84.42
alc R1	0.9 ± 0.1	0.4 ± 0.1 (60% inhibition)	125.51	73.82
alc P1	1.2 ± 0.1	1.2 ± 0.2	100.19	80.04
pro R8	18 ± 5	0.7 ± 0.1 (30% inhibition)	97.37	83.18
pro R5	16 ± 10	0.4 ± 0.1 (60% inhibition)	85.85	92.42
pro R1	16 ± 12	0.4 ± 0.1 (60% inhibition)	108.73	87.67
pro P1	9 ± 5	1.4 ± 0.3	123.06	92.74

Neutrase (neu), Papain (pap), Alcalase (alc), Protamex (pro). Average data ± SD (at least n = 3).

## Conclusions

In the present work, scaled-up enzymatic digestion processes were set up using three different food-grade proteolytic enzymes, followed by physical separation of the peptide fractions. As no chemicals or solvents were used, the end products are completely safe and exploitable for nutraceutical or cosmetic applications. In particular, Protamex and Alcalase treatments produced relatively high amounts of low molecular weight peptides showing, *in vitro*, a range of biological activities. Isolated peptide fractions were demonstrated to possess antioxidant, anti-hypertensive, anti-tyrosinase and/or anti-inflammatory activities, while all were shown to be neither cytotoxic nor irritant. Most of these bioactivities were reported for the first time for this kind of samples. Our results suggest that a future direct exploitation of isolated peptide fractions from an industrial rice by-product in the nutraceutical, functional food and cosmetic industrial fields may be expected. However, depending on the foreseen practical applications, further additional studies have to be carried out to: 1) identify specific active peptides; 2) clarify the connection between amino acid sequence and functional properties; 3) come to a better understanding of the mechanism of absorption and post-absorption modifications in relation to the biological activities, and 4) check the effect of active peptides on the food/cosmetic matrix.
